# PulseNet Lebanon: An Overview of Its Activities, Outbreak Investigations, and Challenges

**DOI:** 10.1089/fpd.2018.2581

**Published:** 2019-07-09

**Authors:** Sukayna M. Fadlallah, Marwa Shehab, Katia Cheaito, Nathaline Haidar-Ahmad, Bassam El Hafi, Majd Saleh, Zeina Nasser, Rima El Hajj, Nada Ghosn, Walid Ammar, Ghassan M. Matar

**Affiliations:** ^1^ Department of Experimental Pathology, Immunology and Microbiology and Center for Infectious Diseases Research, Faculty of Medicine, American University of Beirut, Beirut, Lebanon.; ^2^ Ministry of Public Health, Beirut, Lebanon.; ^3^ Lebanese Agriculture Research Institute, Fanar, Lebanon.

**Keywords:** PulseNet, foodborne, disease, Salmonella, Campylobacter, Listeria, outbreak

## Abstract

***Background:*** Foodborne diseases are still a major health issue in Lebanon, although some steps have been taken forward in food safety. To this purpose, PulseNet Lebanon, a foodborne diseases tracking network, was established in 2009, through the collaboration between the Ministry of Public Health (MoPH) and the American University of Beirut (AUB).

***Materials and Methods:*** Three papers published regarding the PulseNet project were summarized. Initially, clinical and food samples, collected within the surveillance network scope, were identified by using the respective API for *Salmonella* and *Listeria* spp. *Salmonella* spp. were further serotyped by using the Kauffman and White method. *Campylobacter* spp. were determined by the 16 S rRNA sequencing method. Antimicrobial susceptibility to a number of antibiotics was determined by using the disk diffusion method for *Samonella* and *Campylobacter* spp. Genomic diversity was determined by using pulsed field gel electrophoresis (PFGE) and random amplified polymorphic DNA (RAPD).

***Results:*** Results indicated that 290 clinical and 49 food isolates were identified as *Salmonella*. Serotyping revealed the prevalence of ten and seven serotypes in the clinical and food samples, respectively. Fifty-one isolates from chicken ceca and carcass were identified to be *Campylobacter* spp. Fifty-nine samples were identified to be *Listeria monocytogenes*. Antimicrobial susceptibility testing revealed a wide range of resistance among the different samples. PFGE showed a variation in pulsotypes among the *Salmonella* serotypes. PFGE also linked certain outbreaks to their food sources. This method also demonstrated 13 subtypes with 100% similarity among the *L. monocytogenes* isolates. Finally, the *Camplyobcater* spp. were grouped into nine clusters with a minimum similarity of 43.5% using RAPD.

***Conclusion:*** This summary of results shows the importance of implementing a “farm-to-fork” approach in the surveillance of foodborne disease outbreaks in Lebanon, allowing the detection of pathogens causing foodborne disease outbreaks in a timely fashion.

## Introduction

Despite advancements in technology, foodborne diseases remain a global issue that results in health and economic burdens. Sixty-six percent of reported foodborne illnesses are caused by bacteria (Addis and Sisay, 2015). These bacteria include: *Salmonella* spp., *Escherichia coli*, *Listeria monocytogenes*, *Campylobacter* spp., *Clostridium perfringens*, and others (Wolfram, 2017). In 2010, 503 cases of foodborne diseases in Lebanon were identified by clinical laboratories and clustered into 42 episodes of foodborne disease outbreaks, of which *Salmonella* spp. was the most common causative agent. In 2011, the epidemiological surveillance unit at the Ministry of Public Health (MoPH) reported 311 cases of food poisoning and 362 cases of typhoid fever that year, even though salmonellosis is caused by more serotypes than typhoid fever (Unpublished data from MoPH).

In Lebanon, outbreaks are usually detected if spatiotemporal clusters are occurring, or if the exposure history identifies common shared meals (Ghosn *et al.*, 2008). Distributed outbreaks are not detected and are hidden by the endemicity of the disease. Laboratory-based strain surveillance can highly improve surveillance systems of communicable diseases by increasing the specificity of detection (World Health Organization, 2017). Epidemiological surveillance of communicable diseases, including foodborne disease outbreaks, is mandated by law in Lebanon and outbreaks must be reported to the Epidemiological Surveillance Unit at the MoPH in Beirut, Lebanon, on a monthly basis. However, this monthly reporting system delays early detection and data obtained are not useful for timely intervention (Ghosn *et al.*, 2008; World Health Organization, 2012). In addition, undiagnosed cases are common and prevalence data may be biased due to under-reporting. Therefore, the Lebanese chapter of the disease tracking network, PulseNet International, was launched in 2009 in Lebanon between the American University of Beirut (AUB) and the MoPH. This collaboration involves public and private sectors joining forces to reinforce the investigation of foodborne diseases through identifying pathogens causing foodborne disease by time, place, type, subtype, and antimicrobial resistance pattern, and linking clinical cases to their food sources during outbreaks. This will ultimately direct the MoPH in taking the right preventative measures for foodborne diseases. In addition, the knowledge obtained about the antimicrobial resistance patterns will guide physicians in selecting appropriate treatment regimens.

**FIG. 1.**
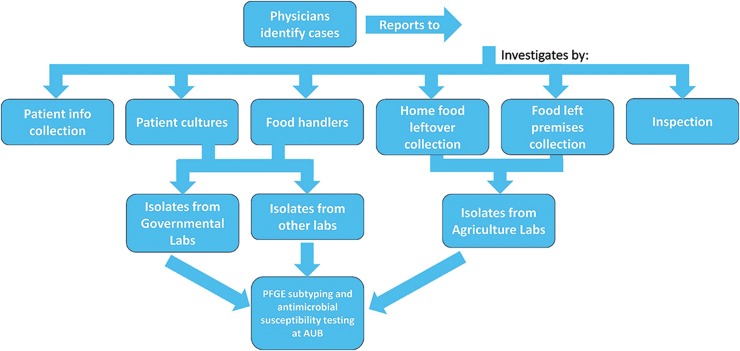
The laboratory-based surveillance and workflow of PulseNet Lebanon.

The main roles of PulseNet Lebanon are to: improve the detection and investigation of foodborne disease (FBD) outbreaks; identify the sources of contamination, vehicles, and dissemination routes; support the recognition and early detection of emerging foodborne pathogens; strengthen the national capabilities for foodborne disease surveillance by facilitating their response capacity, monitoring and investigation of outbreaks; organize and promote training programs and continued education, encouraging the exchange of experience and available resources in the region; promote and strengthen inter-sectorial participation in the formation and functioning of epidemiology surveillance systems; and exchange information with regional networks of PulseNet International. The workflow of PulseNet Lebanon that links physicians, MoPH, laboratory technicians, and public health workers is shown in Figure 1. PulseNet Lebanon is part of the PulseNet MiddleEast, which promotes communication through yearly meetings that are geared by the steering committee (one of which is PulseNet Lebanon) represented by its members. This collaboration ensures exchange of data and materials under the World Health Organization (WHO) umbrella. In addition, PulseNet Lebanon was selected along with Oman to become the certifier of Salmonella pulsed field gel electrophoresis (PFGE) testing.

## Materials and Methods

Three articles publishing work done on *Salmonella* spp., *Camylobacter* spp., and *L. monocytogenes* from food and clinical isolates were summarized to demonstrate some of the research done since PulseNet establishment in Lebanon. The scope of these studies was to identify the circulating species of the bacterial pathogens, determine the antimicrobial resistance pattern, and link food sources to foodborne illness cases during outbreaks. Regular monitoring consists of collecting food samples such as poultry meat (neck and chicken liver), ceca coming from broiler chicken farms and slaughterhouses, and traditionally consumed Lebanese dairy products from randomly selected manufacturers and outlets to screen for the presence of the pathogenic organisms *L. monocytogenes*, *Salmonella* spp., and *Campylobacter* spp. Hence, all samples in the studies were collected within the scope of constant monitoring of foodborne pathogens and each study had its own set of samples isolated within a specific period. In addition, isolates of *L. monocytogenes* and *Salmonella* isolated from food submitted routinely to the laboratory of food microbiology at Lebanese Agriculture Research Institute (LARI) during this period were collected and transferred to the reference laboratory at the Faculty of Medicine at the AUB to be stored for further processing.

Clinical samples were obtained from both governmental and private hospitals and these included around 25 hospitals distributed throughout 5 governorates: Beirut, Mount Lebanon, Nabatieh, Bekaa, and the North. Clinical specimens were submitted to both local laboratories and the reference laboratory and were screened for pathogenic isolates.

### Identification of isolates

At the reference laboratory, isolates were identified to the species level by using respective biochemical kits: For *Salmonella*, API 20E kit (bioMerieux, Marcy L'Etoile, France) and for *Listeria*, API Listeria kit (bioMérieux) were used (Fadlallah *et al.*, 2016; Haidar *et al.*, 2016). Serotyping for the isolates was performed by latex agglutination using mono- and poly-valent anti-sera for O and H antigens according to the Kauffman and White scheme for *Salmonella* isolates. Sequencing was carried out by using the Big Dye Terminator2.0 kit (Applied Biosystems, CA) according to the manufacturer's instruction. Species identification of the *Campylobacter* isolates was determined by using the Basic Local Alignment Search Tool (BLAST) (Fadlallah *et al.*, 2018).

### Antimicrobial susceptibility testing

The antimicrobial susceptibility profiles of the isolates were determined by using the disk diffusion agar method following the 2009 CLSI guidelines for *Salmonella* (Clinical and Laboratory Standards Institute, 2009).

### Genotyping

Genomic relatedness was determined by PFGE on the Bio-Rad CHEF MAPPER (Biorad) using the standard operating procedure for PulseNet PFGE of *E. coli O157:H7*, *E. coli non-O157* (STEC), *Salmonella serotypes*, *Shigella sonnei*, and *Shigella flexneri* with the *Xba*I (Fermentas, Waltham, MA) as restriction endonuclease (Ribot *et al.*, 2006) and a modified PulseNet protocol for *L. monocytogenes* (PulseNet International, 2013) with *Asc*I as restriction endonuclease for the *Salmonella* and *Listeria* isolates, respectively (Fadlallah *et al.*, 2016; Haidar *et al.*, 2016). Regarding the *Campylobacter* isolates, random amplified polymorphic DNA (RAPD) analysis was carried out by using the Ready-To-Go RAPD Analysis Beads Kit (GE, Amersham Place, United Kingdom) as per the manufacturer's instructions. All dendrograms were made by using the UPGMA method (unweighted pair group method using arithmetic averages) and Dice similarity coefficient with the BIONUMERICS software (Fadlallah *et al.*, 2018).

## Results

### Identification of isolates

Between 2011 and 2014, 290 clinical isolates and 49 food samples were identified to be *Salmonella* (Fadlallah *et al.*, 2016). Fifty-nine isolates obtained from local and imported food collected from the Lebanese market were identified to be *L. monocytogenes* during 2012 and 2013 (Haidar *et al.*, 2016). Also within the same period, 51 samples isolated from chicken ceca samples taken during the evisceration process (38 isolates) and whole poultry carcass (13 isolates) were positive for *Campylobacter* spp. Samples were identified to be *Campylobacter coli* by sequencing (Fadlallah *et al.*, 2018). Although *C. coli* is found mainly in pigs, however several studies carried out in Lebanon, Italy, Chile, and Washington showed that this strain is found in chicken carcass and ceca, broiler chicken, turkey breast, and chicken liver respectively (Fernández and Pisón, 1996; Talhouk *et al.*, 1998; Zhao *et al.*, 2001; Pezzotti *et al.*, 2003).

Serotyping indicated that 10 and 7 serotypes were prevalent among the clinical and food isolates respectively. *Salmonella* Typhimurium, *Salmonella* Enteritidis, *Salmonella* Braenderup, *Salmonella* Typhi, *Salmonella* Paratyphi A, *Salmonella* Blockley, *and Salmonella* Newport were the common serotypes among the clinical and food isolates. The clinical isolates included additional serotypes: *Salmonella* London, *Salmonella* Paratyphi B, and *Salmonella* Paratyphi C. The two most common serotypes in both food and clinical isolates were *Salmonella* Enteritidis (clinical = 43.4% and food = 20.4%) and *Salmonella* Typhimurium (clinical, *n* = 29% and food, *n* = 28.5%) (Fadlallah *et al.*, 2016). Table 1 shows the distribution of *Salmonella* serotypes isolated from clinical and food samples.

**Table 1.** Distribution of Salmonella Serotypes Isolated from Clinical and Food Samples (Fadlallah et al., 2016)

*Serotype*	*Total*	
	*Clinical*	*Food*
*Salmonella* Typhimurium	84 (29.0)	14 (28.5)
*Salmonella* Enteritidis	126 (43.4)	10 (20.4)
*Salmonella* Braenderup	21 (7.2)	4 (8.2)
*Salmonella* Typhi	19 (6.6)	3 (6.1)
*Salmonella* London	10 (3.4)	—
*Salmonella* Paratyphi A	8 (2.8)	8 (16.3)
*Salmonella* Blockley	3 (1.0)	2 (4.1)
*Salmonella* Paratyphi B	2 (0.7)	—
*Salmonella* Newport	2 (0.7)	4 (8.2)
*Salmonella* Paratyhi C	2 (0.7)	—
Other	13 (4.5)	4 (8.2)
Total	290 (100)	49 (100)

### Antimicrobial susceptibility testing

Overall, 73.8% of the clinical samples and 75.5% of the food samples were susceptible to the four antibiotics tested: ampicillin, trimethoprim-sulfamethoxazole, ciprofloxacin, and ceftazidime in the *Salmonella* isolates. Regarding the clinical samples, 68 isolates showed resistance to ampicillin, 23 to trimethoprim-sulfamethoxazole, 11 to ciprofloxacin, and 7 to ceftazidime (out of 290 isolates). Among the food strains, 10 isolates showed resistance to ampicillin, 5 to ciprofloxacin, and 1 to ceftazidime (out of 49 isolates). However, they were all susceptible to trimethoprim-sulfamethoxazole (Fadlallah *et al.*, 2016).

### Genotyping

Regarding the *Listeria* isolates, PFGE analysis showed the presence of 13 different subtypes with 100% similarity that were grouped into 6 (A–E) clusters of 90% genomic similarity. The most predominant clusters were E, which had 33 isolates (including subtypes GX6A16.0008, GX6A16.0009, and GX6A16.0010), followed by cluster B, which contained 12 isolates (including subtypes GX6A16.0000, GX6A16.0001, GX6A16.0002, and GX6A16.0003) and cluster D (including subtypes GX6A16.0006 and GX6A16.0007) with 10 isolates. Clustered subtypes were particular to the country of origin: Cluster B contained isolates from Lebanese products only (cheese and raw meat), whereas clusters D and E consisted mostly of Vietnamese fish filet (Haidar *et al.*, 2016).

Regarding the *Salmonella* isolates, PFGE showed a wide range of pulsotypes. *Salmonella* Typhimurium isolates had 13 and 7 pulsotypes recovered from clinical and food samples, respectively. In addition, the same pulsotypes (two) of *Salmonella* Enteritidis were shown to be present in both food and clinical samples. There were five PFGE profiles in the clinical isolates and one in the food isolates in the *Salmonella* Braenderup serotype. On the other hand, *Salmonella* Typhi exhibited four pulsotypes in the clinical samples and three pulsotypes in the food samples (Fadlallah *et al.*, 2016).

Eight pulsotypes of *Salmonella* London were identified in the clinical samples. Further, *Salmonella* Paratyphi A had six pulsotypes within the clinical isolates and eight pulsotypes within the food isolates. Two pulsotypes of *Salmonella* Blockley were isolated from the clinical and food samples. Further, there were four and one pulsotypes of *Salmonella* Newport isolated from food and clinical samples, respectively. Both *Salmonella* Paratyphi B and C had only one pulsotype in the clinical samples (Fadlallah *et al.*, 2016).

RAPD in the *Campylobacter* isolates showed the presence of nine distinct clusters, namely A (8%), B (4%), C (10%), D (6%), E (4%), F (16%), G (6%), H (39%), and I (4%). The most common RAPD type, H, contained 20 isolates that were 55.1% genomically related; the RAPD Type F included 8 isolates having 47.7% genomic similarity; within this cluster, 2 isolates were genetically identical. The RAPD Type C had a genomic relatedness of 45.8% and contained five isolates; the RAPD Type A included four isolates, all of which had a genomic similarity of 45.5%. RAPD Type D and G showed a genomic relatedness of 57.3% and 47.7%, respectively, and contained three isolates. RAPD Type B, E, and I had two isolates each, showing a genomic similarity of 47.1%, 54.5%, and 43.5%, respectively (Fadlallah *et al.*, 2018).

**FIG. 2.**
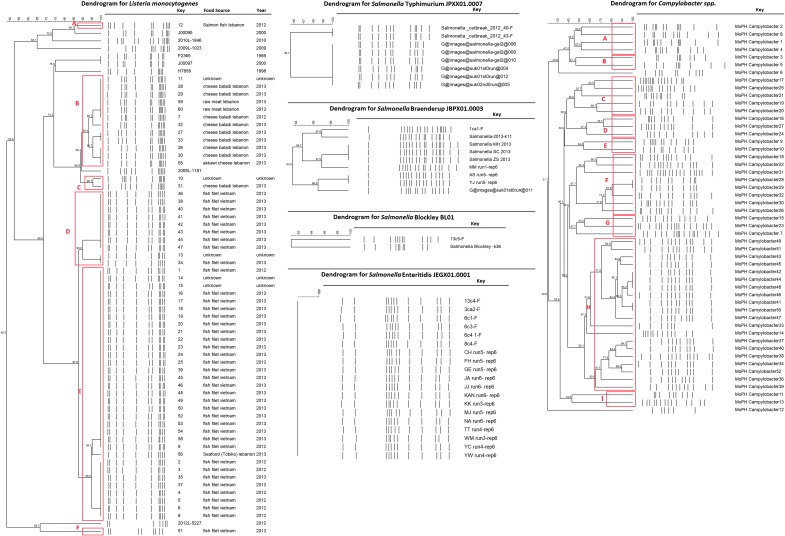
Summary of the dendograms of the genotyping results of the isolates. Images from left to right: summary of the *Listeria* isolates done by PFGE; sample of the *Salmonella* isolates done by PFGE; summary of the *Campylobacter* isolates done by RAPD (Fadlallah *et al.*, 2016, 2018; Haidar *et al.*, 2016). PFGE, pulsed field gel electrophoresis; RAPD, random amplified polymorphic DNA.

Figure 2 shows a summary of the genotyping results.

### Outbreak investigations

During this period, PFGE was also used to link food to clinical isolates during outbreaks. During 2011, two outbreaks of *Salmonella* in different areas of Lebanon were identified. Clinical and suspected food samples were sent to the AUB lab for testing. The first outbreak occurred in Nabatieh during 2011. Five clinical samples were received on February 22, 2011 whereas the suspected food sample (raw meat) was received on March 4, 2011. PFGE and serotyping showed that all clinical samples and the raw meat sample were *Salmonella* Typhimurium with the pulsotype JPXX01. 0002. The second outbreak was identified in Mount Lebanon in September. Eight clinical samples were isolated on September 22, 2011 and two Arabic sweets were identified on October 4, 2011. The causative agent was found out to be *Salmonella* Enteritidis pulsotype, JEGX01.0001 (Fadlallah *et al.*, 2016).

## Conclusion

PulseNet Lebanon has been able during the past 10 years to improve surveillance and issue early warning of foodborne and waterborne outbreaks. In addition, strains of certain bacterial pathogens such as JEGX01.0001 Salmonella Enteritidis that are dominant within the region were identified. New emerging strains were able to be detected through this disease tracking network. Finally, PulseNet Lebanon was able to promote communications between laboratories and public health facilities by carrying out workshops and trainings every year in the different governorates. However, challenges remain, such as miscommunication and limited cooperation of some hospitals with the MoPH, delivery of samples in batches to the PulseNet laboratory, and turn-over of laboratory technicians.
